# Supplementary material for: Reference Gene Selection for RT-qPCR Normalization in Toxoplasma gondii Exposed to Broxaldine
Source: Int J Mol Sci. 2024 Oct 23;25(21):11403. doi: 10.3390/ijms252111403 (PMC11546418; doi:10.3390/ijms252111403)
Supplement: Supplementary file 1 [file ijms-25-11403-s001.zip › ijms-3230564-supplementary.pdf]

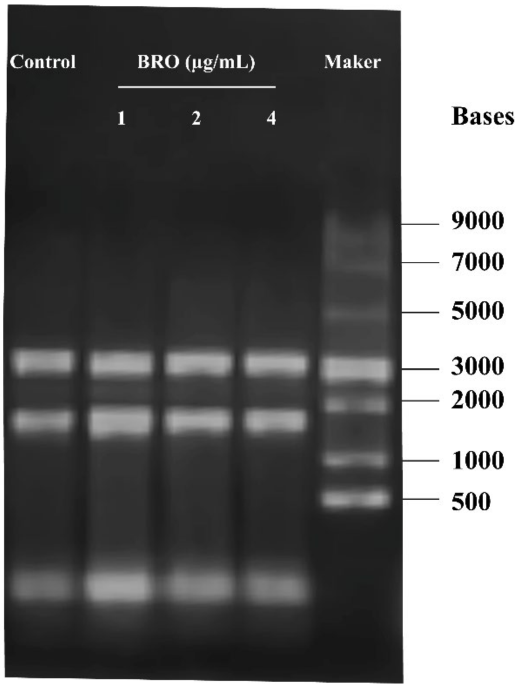

Figure S1. The quality of total RNA was assessed by agarose gel electrophoresis. One sample from each group was randomly selected for this analysis.

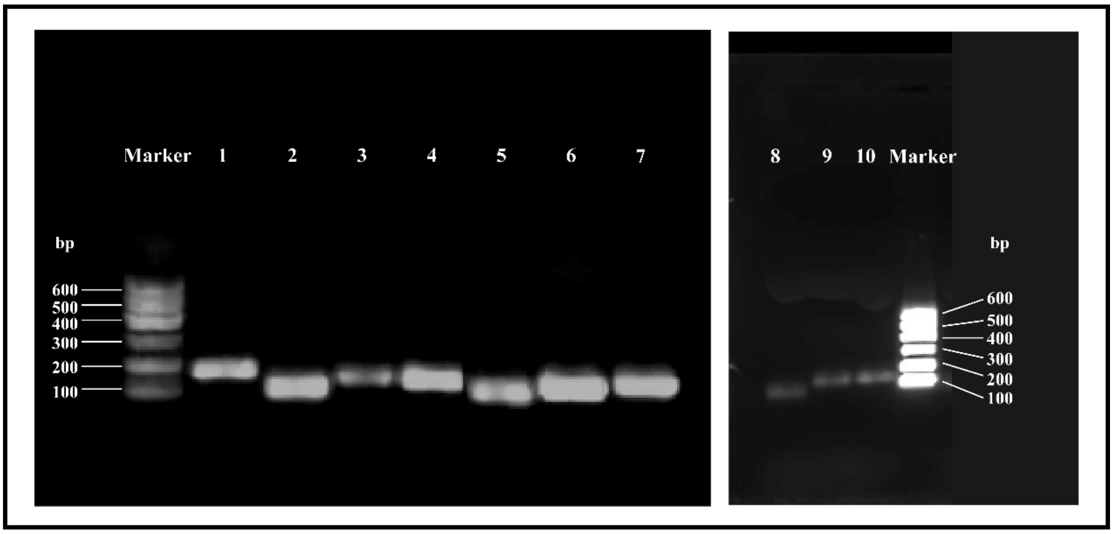

Figure S2. Agarose gel electrophoresis of RT-qPCR products of candidate reference genes. The number represents the following genes. 1: *TGME49_316400*; 2: *TGME49_247220*; 3: *TGME49_235930*; 4: *TGME49_249180*; 5: *TGME49_205470*; 6: *TGME49_209030*; 7: *TGME49_220950*; 8: *TGME49_212300*; 9: *TGME49_289690*; 10: *TGME49_226020*.

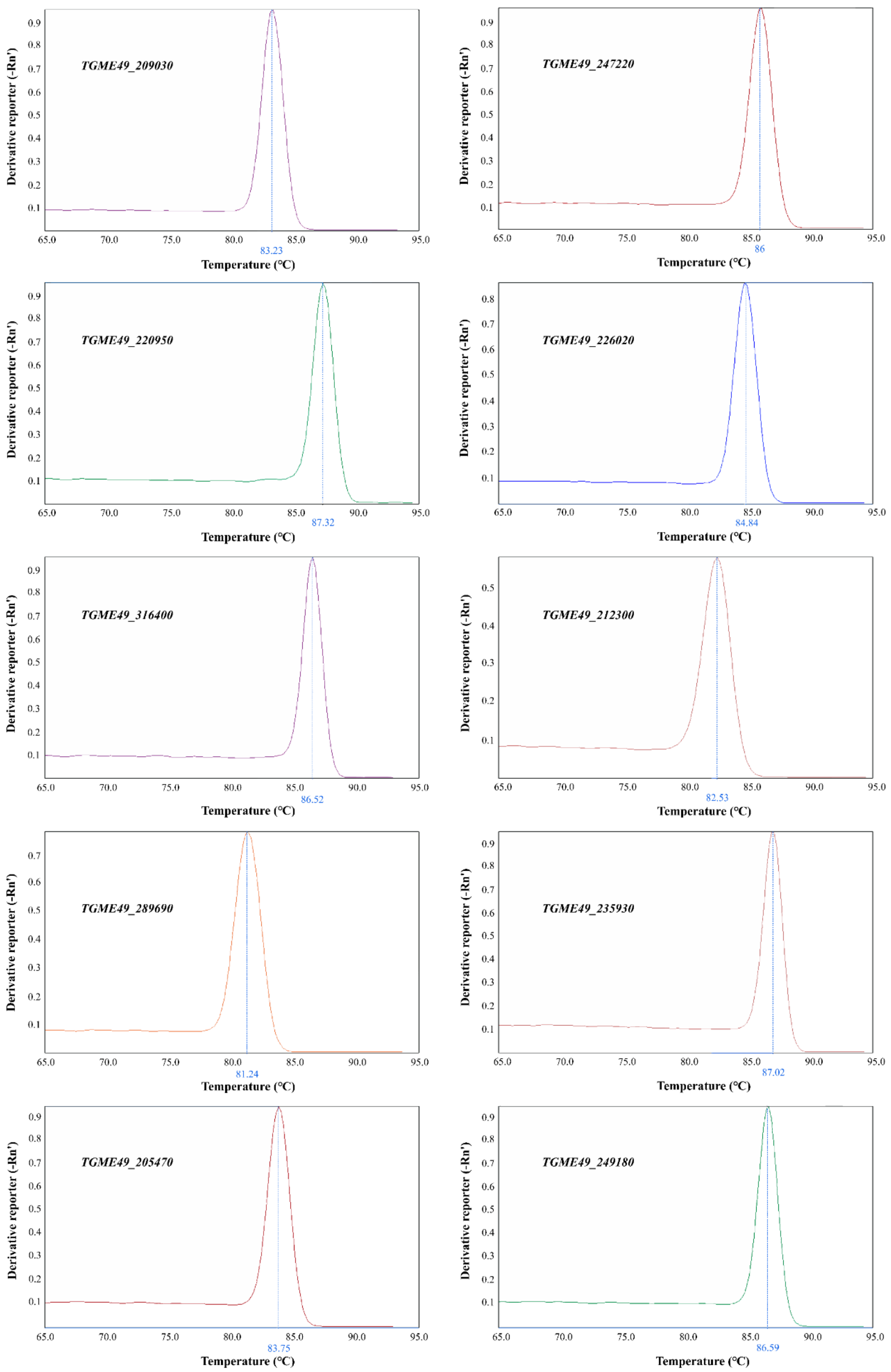

Figure S3. Melting curve of candidate reference genes.

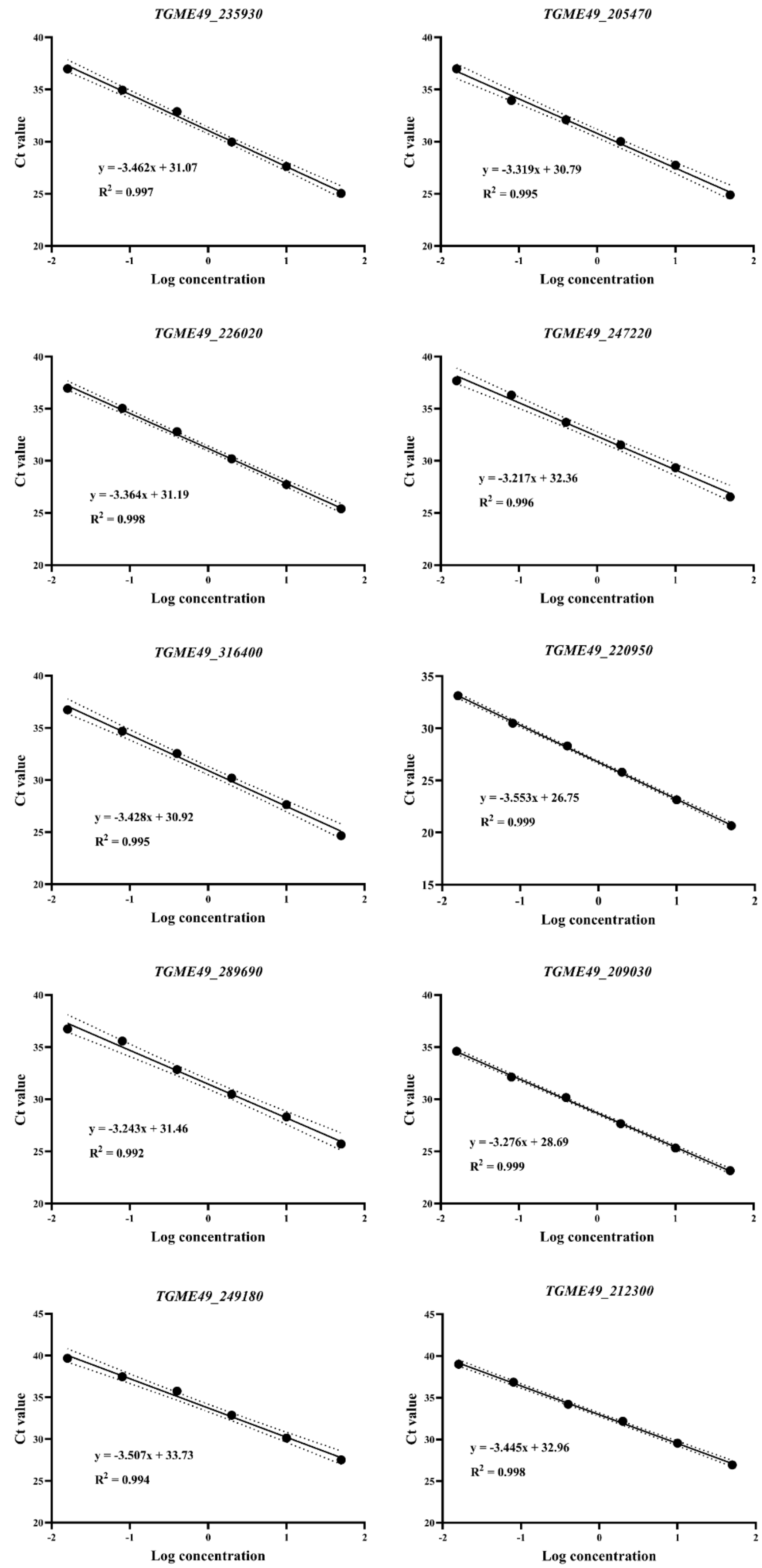

Figure S4. Standard curves of candidate reference genes.

Table S1. Gene primer sequences used for reference gene validation

Gene	Gene description	Primer sequences (5′ →3′)
TGME49_245980	hypothetical protein	F: GTACCAGTGTGACGGCAGAG<br>R: CCGAGAGTAGATGTGGCGTG
TGME49_268970	hypothetical protein	F: ACGGCAGACAAACTTCACCCTTC<br>R: GAATCGGACCAACGGCACTGAG
TGME49_260190	microneme protein *MIC13*	F: GCCAGCATCGAAATGGATTGG<br>R: TAGCGAGCGACACCAGTTTT
TGME49_273130	SAG-related sequence *SRS30A*	F: AACTTGGTGGACGAAAGCCA<br>R: TGAACTGGTGTCTGTCGGTG
TGME49_247520	WAVE complex interacting protein *WIP*	F: GGTCACATTCGAGCCCTACC<br>R: GACCGTAATGATGGGGGCAA
TGME49_252360	rhoptry kinase family protein *ROP24* (incomplete catalytic triad)	F: GCCTACTCCACCACAGGTTC<br>R: CGACCATCAACTGCAAGCAC
ATG8	autophagy-related protein *ATG8*	F: ACGAAGTGTCCTTCGAAAAGAGG<br>R: AGCATGTTCATCGGCACGAG
ATG3	autophagy-related protein 3 atg3, putative	F: CCTACCTGGCAGTGGAAAGG<br>R: GCGAACACGACGGTAACAAG
ATG7	autophagy-related protein *ATG7*	F: GTTGCTTGGGGAGTTAGGGAA<br>R: GAGACACAGCGTCCTCGTAG
DGAT	diacylglycerol acyltransferase	F: GGAAGTGTGCTATCCCTTACAC<br>R: CTCCCTTACCAAAGCCGATAAT
ASH4	serine hydrolase *ASH4*	F: GGCAAGCCTCACCATCATCT<br>R: GCTGTGCCCATATCCGACATA
ACS1	acyl-CoA synthetase *ACS1*	F: TCCCTTTCGTCATGAAACTCA<br>R: GCCGTAAATGGAGTTGATCG
CYP450mt	cytochrome p450	F: TTAAGCAAAAGCGGCGTGTT<br>R: TGAGAACTCGCAACCTCCAC
ATPB	ATP synthase beta subunit *ATP-B*	F: GACACAGGTGCTCCCATTCA<br>R: ACCGCCAAAGAGACCGATTT
ICAP2	ATP synthase F0 subunit b-like protein	F: GGCGTCTTTGCAGGAATTGG<br>R: TGCCTCTGTAGCCTTGACTG
